# Supplementary material for: The Pharmacy of the Future: Pharmacy Professionals’ Perceptions and Contributions Regarding New Services in Community Pharmacies
Source: Healthcare (Basel). 2023 Sep 18;11(18):2580. doi: 10.3390/healthcare11182580 (PMC10531482; doi:10.3390/healthcare11182580)
Supplement: Supplementary file 1 [file healthcare-11-02580-s001.zip › healthcare-2553232-supplementary.pdf]

Table S1. Questionnaire applied to participants

**Sociodemographic questions**					
Age	District				
Gender	Degree				
Years of experience					
**Questionnaire for perception assessment**					
**Items**	**Response Options**				
**Overall, the role of community pharmacies involves:**	Totally Disagree	Disagree	Neither Agree or Disagree	Agree	Totally Agree
Evaluate the patient/client's complaint(s)					
Evaluate therapeutic adhesion.					
Evaluate/Measure physiological parameters					
Refer to a specialized doctor.					
Monitor pharmacological therapy.					
Monitor non-pharmacological therapy.					
Other (please, describe other relevant examples)					
**Do you consider that community pharmacies can provide new ser-vices for the following conditions:**					
In drug-supplement interaction.					
Other (please, describe other relevant examples)					
**Community pharmacies have the potential to include new services given:**					
The geographical proximity that pharmacies have with the community.					
The proximity, in terms of relationship between pharmacy professional and patient/client.					
The existence of physical spaces.					
The existence of human resources.					
The availability of professionals.					

The current knowledge of pharmacy professionals on drug-supplement interaction.					
Other (please, describe other relevant potentialities)					
**The advantages of including these new services for you, as a pharmacy professional would be:**					
Improved adequacy of the services for the patient/client.					
Better follow-up on the health state of the patient/client.					
Further differentiation of pharmacy professionals					
Better payment for new services provided.					
The possibility of working in a multidisciplinary team					
Other (please, describe other advantages that you consider relevant)					
**The advantages of including these new services for pharmacies would be:**					
Increased perception of the utility of pharmacies by patients/clients, and the availability of more services.					
Increased profits.					
Prestige.					
Social function.					
Resource optimization.					
Increased competitiveness of pharmacies.					
Other (please, describe other advantages that you consider relevant)					
**The advantages of including these new services for other healthcare professionals would be:**					
Reduced workload.					
Increased intervention success.					
Assuring therapeutic follow-up.					
Gaining additional detailed information about the patient/client.					
Other (please, describe other advantages that you consider relevant)					
**The advantages of including these new services for patients/clients would be:**					
Reducing the number of trips to healthcare services					

Preventing the aggravation of the clinical condition					
Close relationship between patient/client and pharmacy professional					
More confidence in the services provided.					
Faster intervention.					
Optimizing patient/client time in accessing the pharmacy's service.					
Other (please, describe other advantages that you consider relevant)					
**The role of the pharmacy in drug-supplement interaction would imply assessing:**					
Previously prescribed drugs.					
Supplement consumption.					
The risk of drug-supplement interactions that the patient/client plans to acquire.					
Glycaemia.					
Cholesterol.					
Triglycerides.					
Weight/height.					
Blood pressure.					
Other (please, describe other parameters that you consider relevant).					
**The role of pharmacies in non-pharmacological therapeutics involves/should involve monitoring:**					
Physical activity.					
Lifestyle.					
Food diet.					
Smoking cessation.					
Sleep hygiene.					
Other (please, describe other parameters that you consider relevant).					
**The conditions for implementation of new services in pharmacies are/should be:**					
Clarifying legal requisites, namely about the data that can be shared.					

Pharmacy professionals' training, for example, more knowledge about medical conditions/pathologies.					
Specialization of pharmacy professionals in new conditions to be considered, particularly in drug-supplement interaction.					
Recruitment of new specialized professionals for pharmacies.					
Adequate physical space/pharmacy facilities.					
**For drug-supplement interactions:**					
Adequate equipment and computer systems at pharmacies for drug-supplement interaction.					
Printer for printing a list of individual interactions to give patients/clients.					
Interface with access to the data collected by the pharmacy professional.					
Data bank with drug-supplement interactions.					
Interface for other healthcare professionals that do not work in a pharmacy.					
Alerts for herb interactions for various healthcare professionals involved in the monitoring, such as doctors, nutritionists, and other pharmacy professionals, giving scientific knowledge.					
Platform for sharing valid scientific information to support interaction alerts.					
Other (please, describe other parameters that you consider relevant).					
**The new services made available at community pharmacies, for drug-supplement interactions, should be financially supported by:**					
The pharmaceutical industry.					
The pharmacy itself.					
The state.					
Insurance companies.					
Patient/Client.					
Other (please, describe other examples that you consider relevant).					
**The difficulties in implementing new services in pharmacies are/could be:**					
More workload for pharmacy professionals.					

More financial investment by the pharmacy.					
More time investment by pharmacy professionals.					
The sensitivity of the data managed, namely the sharing of information between pharmacy professionals and healthcare professionals.					
The sensitivity of the data managed, namely the sharing of information between pharmacies.					
Lack of training about the use of new equipment.					
Lack of training about how the new services work.					
Lack of training for pharmacy professionals to act in drug-supplement interactions.					
Lack of acceptance by patients/clients about possible drug-supplement interactions that they take or plans to take.					
Absence of a regulatory entity for supplements.					
No knowledge about the acquisition of drugs or supplements acquired in other spaces, or at other pharmacies					
Other (please, describe other examples that you consider relevant).					
**Do you consider that the use new services can/could be activated:**					
With the exacerbation of the problem.					
With the change in therapy.					
In the beginning of the therapy.					
On medication dispensing.					
When there is a patient/client complaint.					
When an adverse reaction occurs.					
When the disease is not under control.					
With bad device use.					
Other (please, describe other examples that you consider relevant).					
**Can promote patient/client adherence to the new service, like:**					
Providing discounts for the new services.					

The new services being free of charges.					
Assuring the co-payment for the new services.					
Assuring the dissemination of the added value of the new services.					
Assuring the confidentiality in sharing clinical information.					
Other (please, describe other examples that you consider relevant).					
**How would you classify the adherence of the different stakeholders to these new services:**					
By pharmacy professionals.					
By healthcare professionals, including doctors, nutritionists, among others.					
By patients/clients.					
Your personal adherence.					
In case you want to give a comment/suggestion, please, use this space.					
